# Supplementary material for: Automatic and accurate ligand structure determination guided by cryo-electron microscopy maps
Source: Nat Commun. 2023 Mar 1;14:1164. doi: 10.1038/s41467-023-36732-5 (PMC9976687; doi:10.1038/s41467-023-36732-5)
Supplement: Supplementary file 1 — Supplementary Information [file 41467_2023_36732_MOESM1_ESM.pdf]

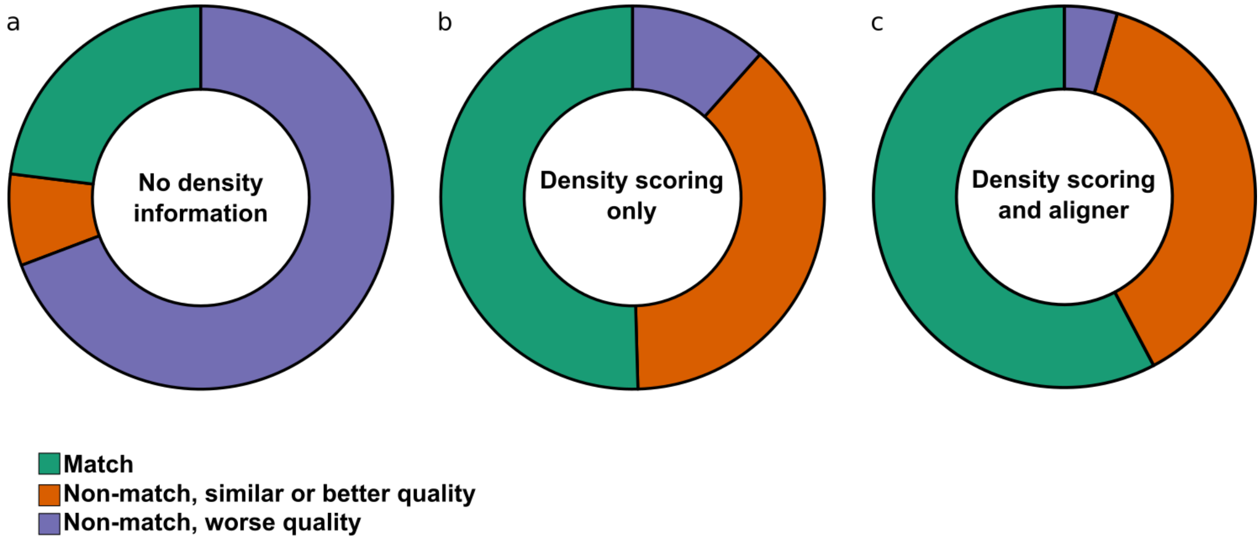

Supplementary Figure 1. Docking results with varying density information. Docking results when docked with no density information in the genetic algorithm (a) and with density scoring evaluation during the genetic algorithm, but not during sampling (b). (a) Without density information, ligand docking can produce a model within 1 Å RMSD to the deposited model for only 23% of cases, and models often do not fit the density, having a worse density correlation or fewer hydrogen bonds for 69% of cases. (b) Adding a density fit score during GA evaluation greatly improves modeling success, getting 50% of cases within 1 Å RMSD to the deposited structure, but is still lower than when density information is included in sampling (c).

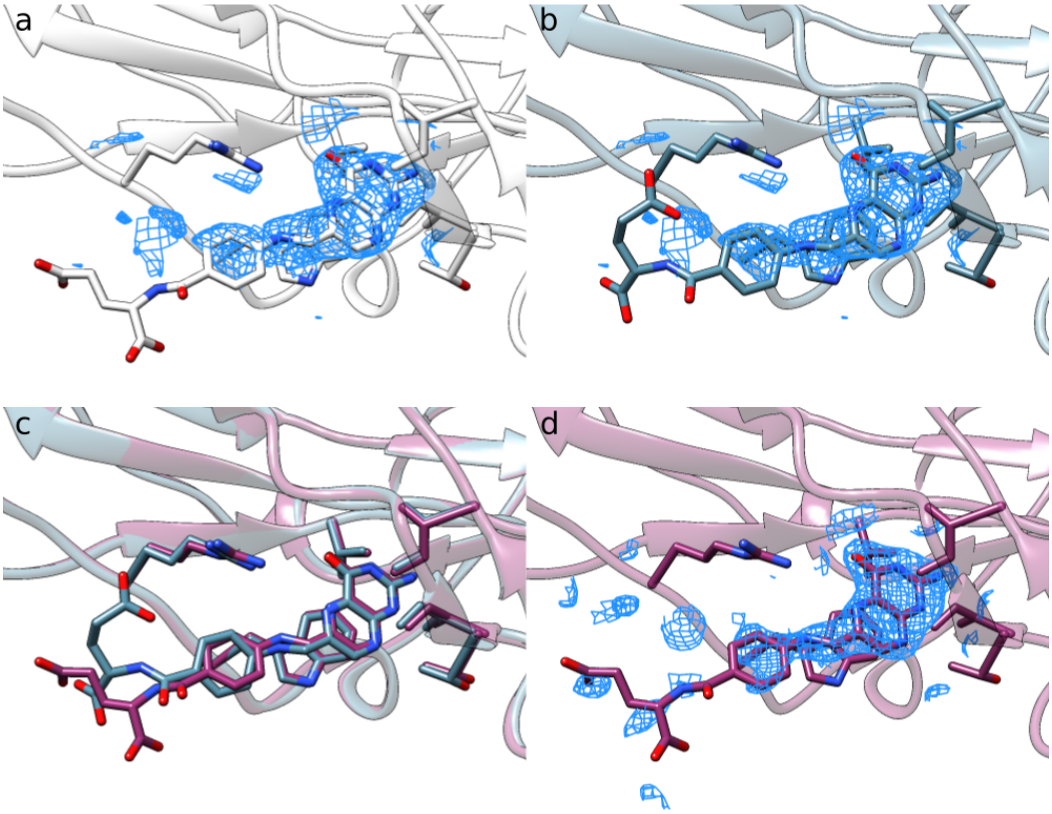

Supplementary Figure 2. Ligand models of folate in MERS-CoV. (a,b) The deposited model (EMDB: 23674, PDB: 7M5E, local resolution: 3.81 Å) (a) and docked model (b) are similar in ordered regions of the ligand with strong cryoEM density and vary in the solvent exposed unordered region. (c) A superposition of the docked model (blue) and crystal model (purple) (PDB: 5VYH) reveals alignment where the ligand is interacting with the receptor but disagreement in the unordered region. (d) The crystal model shown with its 2mFo-Fc map (contour = 1σ) shows weak density in the solvent exposed region.

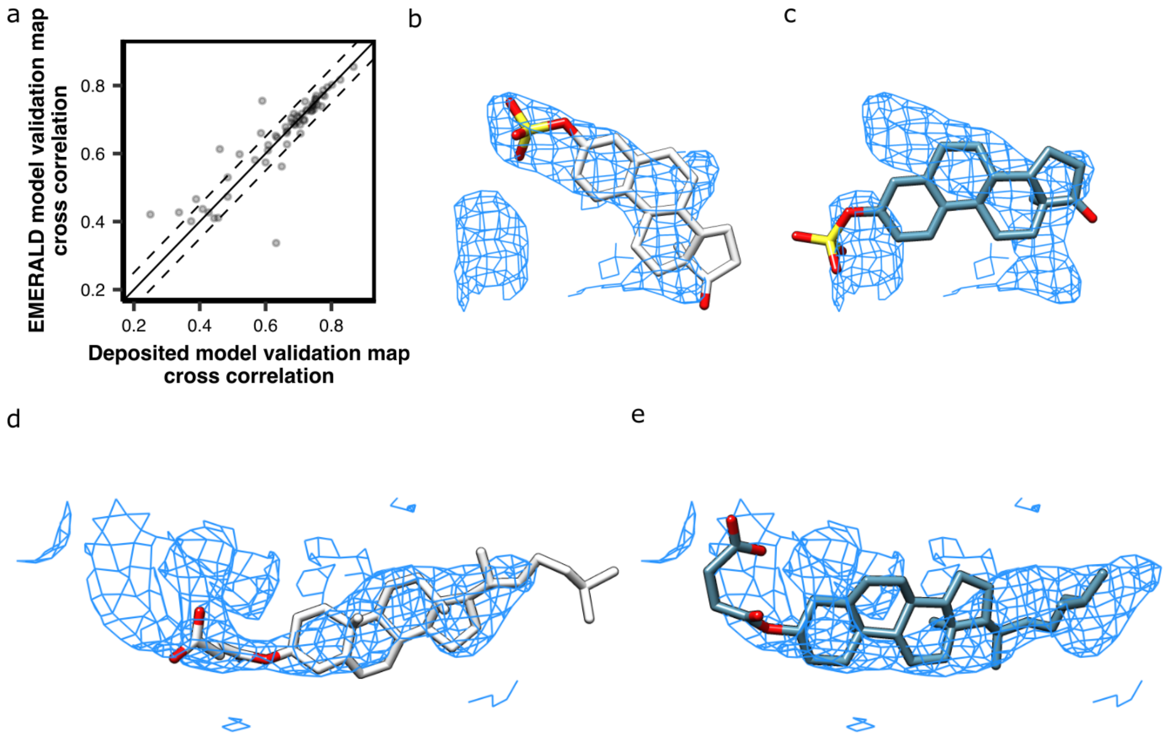

Supplementary Figure 3. Validation of models by calculating map correlation to half maps. (a) Comparison of the map correlation of the deposited model and EMERALD model to a half map. Dashed lines are at +/- 0.05 correlation values above or below equal values. Points above the dashed line are likely cases where the EMERALD model fits better than the deposited (shown in Fig. 4c, d). Points below the bottom dashed line are likely cases where the EMERALD model is worse than the deposited model. (b-e) Two cases where the EMERALD model was counted as similar or better than the deposited model, but is not by half map analysis. Deposited (b) and EMERALD (c) model of estrone 3-sulfate in ABCG2 (EMDB: 12939, PDB: 7OJ8, local resolution: 3.59 Å). Deposited (d) and EMERALD (e) model of cholesterol hemisuccinate in PfCRT (EMDB: 20806, PDB: 6UKJ, local resolution: 3.21 Å).

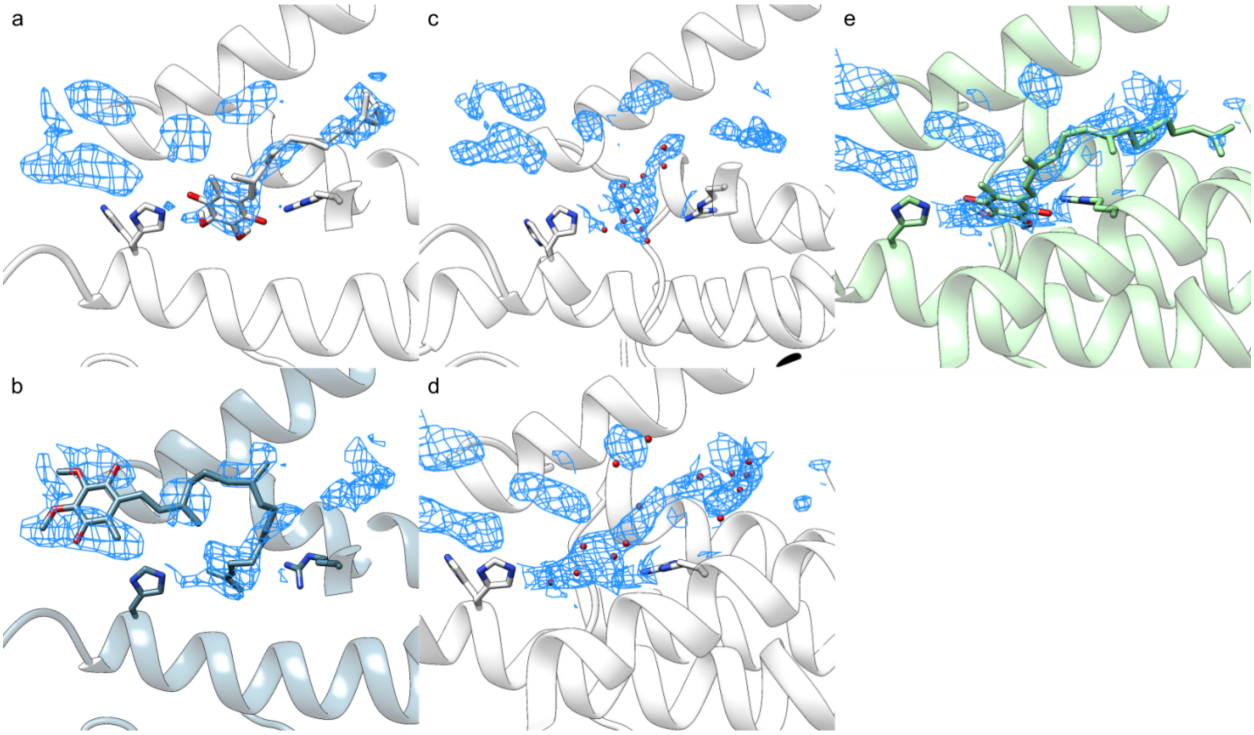

Supplementary Figure 4. Low-pass filtered map leads to a more complete skeleton and produces a model similar to the deposited model. (a,b) Ubiquinone binding site in cytochrome bo3 in the deposited model (EMDB: 30475, PDB: 7CUW, local resolution: 2.74 Å) (a) and docked model (b). EMERALD cannot find the known binding conformation and places the ligand in noisy density. (c) The density skeleton (red spheres) determined by our erosion protocol only includes density for a small portion of the ligand. (d) When the EM map is low-pass filtered at a 4Å cutoff, the density is more continuous, and the skeleton covers all of the ligand density. (e) The lowest-energy model from EMERALD after the map processing has a similar density correlation and similar hydrogen bonds as the deposited model.

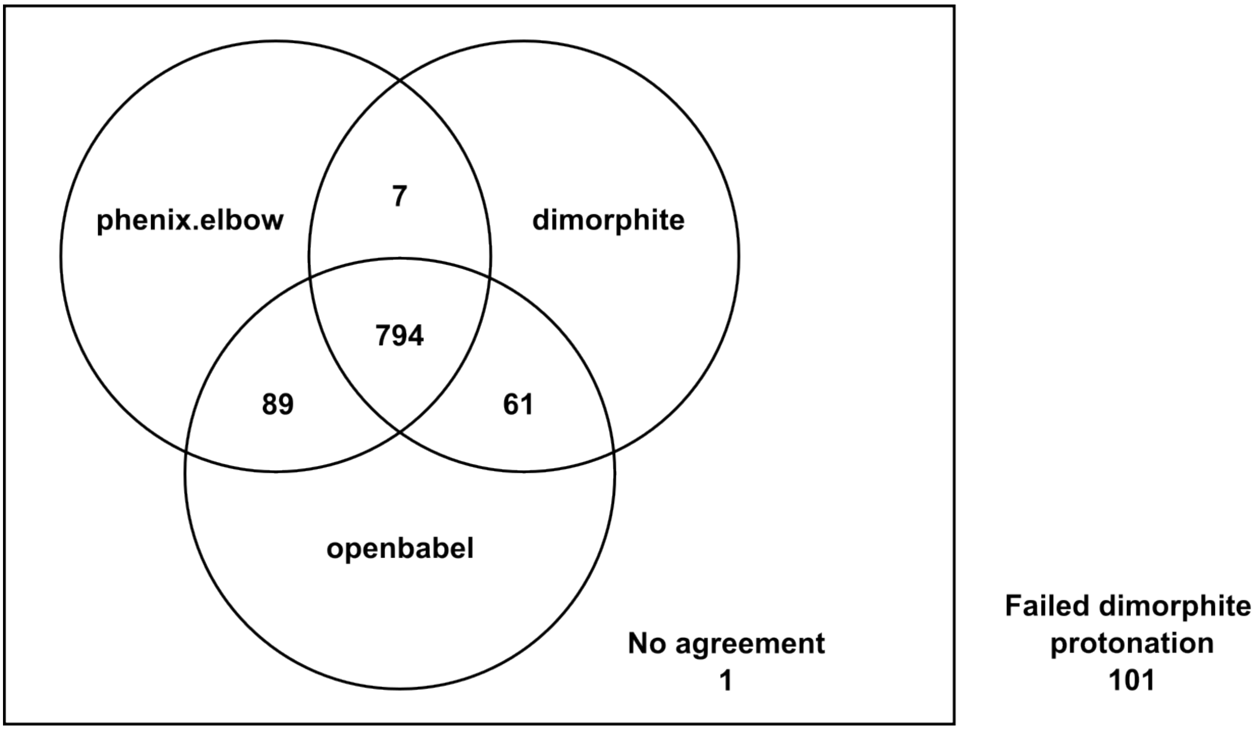

Supplementary Figure 5. Agreement of protonation state assignments. Three protonation assignment methods were used to determine the protonation state for a small molecule in EMERALD. The state assigned by 2 or more methods was used for docking.
