# Supplementary material for: Automatic and accurate ligand structure determination guided by cryo-electron microscopy maps
Source: Nat Commun. 2023 Mar 1;14:1164. doi: 10.1038/s41467-023-36732-5 (PMC9976687; doi:10.1038/s41467-023-36732-5)
Supplement: Supplementary file 3 — Description of Additional Supplementary Files [file 41467_2023_36732_MOESM3_ESM.pdf]

Description of Additional Supplementary Files

File Name: Supplementary Data 1
Description: Models used in main figures. PDB files of EMERALD docked models with hydrogen bonds for every model in Figs. 3-6.

File Name: Supplementary Data 2
Description: EMERALD Demo. Scripts and input files needed to run EMERALD docking on an example. A version of Rosetta with EMERALD code (v2023.06 or later) is needed for the demo.
