# Supplementary material for: Automatic and accurate ligand structure determination guided by cryo-electron microscopy maps
Source: Nat Commun. 2023 Mar 1;14:1164. doi: 10.1038/s41467-023-36732-5 (PMC9976687; doi:10.1038/s41467-023-36732-5)
Supplement: Supplementary file 6 — Reporting Summary [file 41467_2023_36732_MOESM6_ESM.pdf]

Corresponding author(s): Frank DiMaio

Last updated by author(s): Jan 27, 2023

Reporting Summary

Nature Portfolio wishes to improve the reproducibility of the work that we publish. This form provides structure for consistency and transparency in reporting. For further information on Nature Portfolio policies, see our Editorial Policies and the Editorial Policy Checklist.

Statistics

For all statistical analyses, confirm that the following items are present in the figure legend, table legend, main text, or Methods section.

n/a	Confirmed	
☐	☒	The exact sample size (*n*) for each experimental group/condition, given as a discrete number and unit of measurement
☒	☐	A statement on whether measurements were taken from distinct samples or whether the same sample was measured repeatedly
☒	☐	The statistical test(s) used AND whether they are one- or two-sided<br>*Only common tests should be described solely by name; describe more complex techniques in the Methods section.*
☒	☐	A description of all covariates tested
☒	☐	A description of any assumptions or corrections, such as tests of normality and adjustment for multiple comparisons
☐	☒	A full description of the statistical parameters including central tendency (e.g. means) or other basic estimates (e.g. regression coefficient) AND variation (e.g. standard deviation) or associated estimates of uncertainty (e.g. confidence intervals)
☒	☐	For null hypothesis testing, the test statistic (e.g. *F*, *t*, *r*) with confidence intervals, effect sizes, degrees of freedom and *P* value noted<br>*Give P values as exact values whenever suitable.*
☒	☐	For Bayesian analysis, information on the choice of priors and Markov chain Monte Carlo settings
☒	☐	For hierarchical and complex designs, identification of the appropriate level for tests and full reporting of outcomes
☒	☐	Estimates of effect sizes (e.g. Cohen's *d*, Pearson's *r*), indicating how they were calculated

Our web collection on statistics for biologists contains articles on many of the points above.

Software and code

Policy information about availability of computer code

Data collection	Rosetta (v2023.06 or later)<br>phenix.auto_sharpen (v. dev-4761)<br>MonoRes via Xmipp
Data analysis	Rosetta (v2023.6 or later)<br>UCSF Chimera (v. 1.15)<br>R (v. 4.1.2)<br>ggplot2 (v. 3.3.6)<br>phenix.elbow (v. 1.14)<br>openbabel (v. 3.1.0)<br>dimorphite (v. 1.2.4)<br>RDKit (v. 2020.09.1.0)

For manuscripts utilizing custom algorithms or software that are central to the research but not yet described in published literature, software must be made available to editors and reviewers. We strongly encourage code deposition in a community repository (e.g. GitHub). See the Nature Portfolio guidelines for submitting code & software for further information.

Data

Policy information about availability of data

All manuscripts must include a data availability statement. This statement should provide the following information, where applicable:

- Accession codes, unique identifiers, or web links for publicly available datasets
- A description of any restrictions on data availability
- For clinical datasets or third party data, please ensure that the statement adheres to our policy

Models with hydrogen atoms for EMERALD-docked models in all main figures (Figs. 3-6) are provided in Supplementary Data 1. Source data for Figures 2B, 2C, and Supplementary Figure 3A are provided with the paper. The lowest energy models for all cases for each individual EMERALD run are available for download at https://files.ipd.uw.edu/pub/EMERALD/EMERALD_top1_models.tar.gz [https://files.ipd.uw.edu/pub/EMERALD/EMERALD_top1_models.tar.gz]. PDB accession codes used in this manuscript are: 7LEP [http://doi.org/10.2210/pdb7LEP/pdb], 5ZG2 [http://doi.org/10.2210/pdb5ZG2/pdb]), 7OCE [http://doi.org/10.2210/pdb7OCE/pdb], 6FQH [http://doi.org/10.2210/pdb6FQH/pdb]), 7LRD [http://doi.org/10.2210/pdb7LRD/pdb], 7KWE [http://doi.org/10.2210/pdb7KWE/pdb]), 6W6E [http://doi.org/10.2210/pdb6W6E/pdb], 5LJ8 [http://doi.org/10.2210/pdb5LJ8/pdb]), 7OCF [http://doi.org/10.2210/pdb7OCF/pdb], 3TKD [http://doi.org/10.2210/pdb3TKD/pdb], 6VKS [http://doi.org/10.2210/pdb6VKS/pdb], 6NR2 [http://doi.org/10.2210/pdb6NR2/pdb], 7BSP [http://doi.org/10.2210/pdb7BSP/pdb], 6X5C [http://doi.org/10.2210/pdb6X5C/pdb], 6WLW [http://doi.org/10.2210/pdb6WLW/pdb], 7CKZ [http://doi.org/10.2210/pdb7CKZ/pdb]. EMDB accession codes used in this manuscript are: 23292 [https://www.ebi.ac.uk/pdbe/entry/emdb/EMD-23292], 12805 [https://www.ebi.ac.uk/pdbe/entry/emdb/EMD-12805], 23495 [https://www.ebi.ac.uk/pdbe/entry/emdb/EMD-23495], 21553 [https://www.ebi.ac.uk/pdbe/entry/emdb/EMD-21553], 12806 [https://www.ebi.ac.uk/pdbe/entry/emdb/EMD-12806], 21228 [https://www.ebi.ac.uk/pdbe/entry/emdb/EMD-21228], 0487 [https://www.ebi.ac.uk/pdbe/entry/emdb/EMD-0487], 30163 [https://www.ebi.ac.uk/pdbe/entry/emdb/EMD-30163], 22049 [https://www.ebi.ac.uk/pdbe/entry/emdb/EMD-22049], 21844 [https://www.ebi.ac.uk/pdbe/entry/emdb/EMD-21844], 30395 [https://www.ebi.ac.uk/pdbe/entry/emdb/EMD-30395].

Human research participants

Policy information about studies involving human research participants and Sex and Gender in Research.

Reporting on sex and gender	N/A
Population characteristics	N/A
Recruitment	N/A
Ethics oversight	N/A

Note that full information on the approval of the study protocol must also be provided in the manuscript.

Field-specific reporting

Please select the one below that is the best fit for your research. If you are not sure, read the appropriate sections before making your selection.

[x] Life sciences [ ] Behavioural & social sciences [ ] Ecological, evolutionary & environmental sciences

For a reference copy of the document with all sections, see nature.com/documents/nr-reporting-summary-flat.pdf

Life sciences study design

All studies must disclose on these points even when the disclosure is negative.

Sample size	No sample sized calculation was performed. EMERALD was benchmarked on as many ligand-bound cryoEM structures as possible, with exclusions listed below. All ligand-bound structures solved by cryoEM were examined for inclusion in our dataset. Since this includes all deposited cases that our method would be applicable to solving, we believe the final size of 1053 cases is a sufficient size of structures to test.
Data exclusions	Several cases were excluded from the tested dataset. First, ligands that were ions or glycans were excluded because of the specificity needed to accurately model. Ligands with 2 or more ligands within the same binding pocket were also excluded as the issue of modeling 2 small molecules simultaneously presets its own challenges outside the scope of the paper. Finally, cases where the macromolecule-ligand PDB structure had a map correlation below 0.4 or the map had regions of unmodeled protein density were excluded to eliminate incomplete or misaligned models. Some cases failed during ligand processing and were excluded from the final dataset as well.
Replication	The same docking protocol was used for all cases. EMERALD was ran on each case 3 times on different seeds to use as confidence for docking results. Example protocols and scripts are included in the methods for reproducibility.
Randomization	Randomization was not relevant to the study. There is only a single test group needed to benchmark the method.
Blinding	Blinding was not relevant to the study. There is only a single test group needed to benchmark the method.

Reporting for specific materials, systems and methods

We require information from authors about some types of materials, experimental systems and methods used in many studies. Here, indicate whether each material, system or method listed is relevant to your study. If you are not sure if a list item applies to your research, read the appropriate section before selecting a response.

Materials & experimental systems

n/a	Involved in the study
☒	☐ Antibodies
☒	☐ Eukaryotic cell lines
☒	☐ Palaeontology and archaeology
☒	☐ Animals and other organisms
☒	☐ Clinical data
☒	☐ Dual use research of concern

Methods

n/a	Involved in the study
☒	☐ ChIP-seq
☒	☐ Flow cytometry
☒	☐ MRI-based neuroimaging
